# Internet-Based Prevention of Re-Victimization for Youth with Care Experience (EMPOWER-YOUTH): Results of a Randomized Controlled Trial

**DOI:** 10.3390/ijerph21091159

**Published:** 2024-08-30

**Authors:** Birgit Wagner, Olivia Lucia Marie Emmerich, Betteke Maria van Noort

**Affiliations:** Department of Psychology, MSB Medical School Berlin, 14197 Berlin, Germany; lucia.emmerich@medicalschool-berlin.de (O.L.M.E.); betteke.van-noort@medicalschool-berlin.de (B.M.v.N.)

**Keywords:** youth with care experience, foster care, victimization, prevention, internet-based, PTSD, depression

## Abstract

Traumatic experiences and maltreatment are highly prevalent among adolescents in foster or institutional care and have severe long-term effects on mental health. Childhood maltreatment increases the risk of revictimization. This study aimed to evaluate the efficacy of the internet-based prevention program EMPOWER YOUTH in reducing victimization experiences among youth with care experience. A total of 163 youth, that is, adolescents in foster or institutional care, adopted adolescents, or young care leavers aged 14 to 21 years (*M_age_* = 17.68 years; *SD* = 2.11) were randomized to the six-module guided program or a wait-list control group. The primary endpoint was the decrease in victimization experiences at an 18-week follow-up. Secondary endpoints included risk perception, aggressive tendencies, empathy, prosocial behavior, depressiveness, post-traumatic stress symptoms, and loneliness. About half of the youth exhibited increased psychopathology. No significant interaction between time-point and group was found for victimization measures, though both groups saw a significant reduction over time (*p* = 0.012, η_p_^2^ = 0.06; *p* = 0.017, η_p_^2^ = 0.06). The intervention group showed a significant increase in perception of thrill-seeking and rebellious risks (*p* = 0.036, η_p_^2^ = 0.04; *p* = 0.026, η_p_^2^ = 0.05). While EMPOWER YOUTH effectively increased risk perception of thrill-seeking and rebellious behaviors, it should be considered an add-on to more intensive interventions for common mental health disorders in this vulnerable group, rather than a stand-alone online program.

## 1. Introduction

The worldwide estimate of children residing in institutional care stands at approximately 5 million, and within this figure, roughly 1 million children reside in Europe [1]. Accurate statistics regarding the global number of children living in foster care or adoptive families are currently lacking. In Germany, the foster care system accommodates approximately 86,000 children and adolescents, while an additional 121,000 children and adolescents reside in institutions [2].

Children and adolescents that are placed in care often experience victimization in the form of neglect, emotional abuse, physical abuse, or sexual abuse. A study from Norway reports a three- to four-fold higher rate of neglect, experiences of violence, or sexual abuse among adolescents in foster care compared to children and adolescents from the general population [3]. Childhood abuse and neglect are associated with mental health problems later in life, such as anxiety, post-traumatic stress symptoms, depression, substance abuse, and sexualized behavior [3,4,5,6,7].

Besides the increased risk for long-term mental health problems, earlier victimization experiences also increase the risk for future victimization experiences [8,9]. Potential mechanisms for this increased revictimization risk could be that, for those placed in care, insecure or disorganized attachment behaviors are more commonly observed, which can lead to interpersonal difficulties [9,10] in combination with low self-esteem and impaired emotion regulation [10]. Moreover, increased threat of harm perception associated with post-traumatic stress symptoms is found to be a significant mechanism through which past victimization predicts revictimization [9]. This is in line with similar findings suggesting that impaired risk recognition increases the likelihood of revictimization [8,11].

Considering the heightened risk of future victimization among young people with care experience, there is a critical demand for a readily accessible intervention tailored to this high-risk age group. In general, few interventions are available for care-experienced youth, and those that are available mostly target caregivers in the care system instead of youth directly [12,13]. However, interventions targeting risk factors and mechanisms for different forms of revictimization are particularly scarce, specifically for care-experienced youth. Recently developed face-to-face interventions targeting youth specifically primarily focus on educational outcomes [14] or psychosocial outcomes, such as employment and delinquency [15].

The objective of the current study is to evaluate the efficacy of a newly developed internet-based prevention program called EMPOWER YOUTH, consisting of six modules, in reducing victimization experiences among youth with care experience [16]. The primary endpoint is the decrease in the number of victimization experiences at an 18-week follow-up. Secondary endpoints include risk perception, aggressive tendencies, empathy, prosocial behavior, depressiveness, post-traumatic stress symptoms and loneliness at the follow-up. It is hypothesized that this prevention program will prove effective in decreasing the occurrence of victimization, enhancing coping strategies in challenging social situations, increasing awareness of risky or dangerous situations, and reducing secondary mental health symptoms.

## 2. Materials and Methods

### 2.1. Study Design and Recruitment

This study is part of a research consortium called EMPOWERYOU, comprising the following universities and institutions (in alphabetical order): Karlsruhe Institute of Technology, Medical School Berlin, PFAD e.V. Association of Foster Care and Adoptive Families in Germany, University of Aachen, University of Bielefeld, and University of Bremen. Intervention effects were assessed through an RCT registered in the German Clinical Trials Register under DRKS00024749. The RCT compared the prevention program with a waiting control group, using an unblinded 1:1 allocation ratio. The wait-list control group received the possibility to complete the intervention program after data collection was completed. Data collection occurred from September 2021 through April 2024 through the online platform empower-youth.de. Before commencing the RCT, a study protocol in accordance with the SPIRIT guidelines [17] was published [16].

Participants were recruited through the organization PFAD e.V. Association of Foster Care and Adoptive Families in Germany, as well as other youth in care-focused organizations, collectively reaching more than 13,000 foster families and young care leavers. Recognizing that many youth with care experience are not affiliated with specific groups or organizations, online recruitment was conducted through social media platforms such as Instagram, Facebook, and TikTok. Additionally, flyers and posters were distributed to child welfare services and other relevant institutions working with care-experienced youth.

Prior to participating in the study, all interested individuals completed an informed consent form. This form explicitly stated that participants could withdraw from the study at any time without providing a reason, and it adhered to the principles of the Declaration of Helsinki. For participants below the age of 16 years, additional informed consent from a legal guardian was obtained. Assessments occurred at two points: before randomization (baseline) and at a follow-up 18 weeks after baseline (i.e., approximately 12 weeks after the last program module). During the initial assessment, participants were blind to their group allocation. Randomization took place after the baseline assessment, conducted by personnel at the University Hospital Aachen, a member of the EMPOWERYOU consortium, who were not otherwise involved in this trial. Hereby, a blind randomization process was ensured. Participants were randomly assigned following a fixed block design of 10 into either the intervention group or the waiting-list control group.

For each online assessment, the participants received an email invitation containing an individual identification code. This coding system ensured anonymous data collection without any direct identifiers. Participants received financial compensation of €70 in the form of a voucher upon completing the second assessment.

Participants were granted the option to withdraw from the study at any time. Moreover, upon request, all accumulated data, including information from the assessments as well as the online program, can be deleted. The principal investigator retained the right to discontinue the intervention for a participant under specific circumstances: (1) in the event of adverse events or serious adverse events, particularly those that involved acute child endangerment or the manifestation of suicidal tendencies, and (2) when there existed an unacceptable benefit-to-risk ratio for the participant. Should a family member or guardian report adverse experiences on behalf of a participant during the trial, established ethical guidelines for participant well-being were adhered to and participants would have received referrals to appropriate support services. In instances where immediate child endangerment was identified, the relevant authorities would have been promptly informed of the situation. Additionally, recommendations from the Data Safety Monitoring Board (DSMB) were followed and study participation might have been suspended for this person. Nonetheless, the participants would retain the option to complete the prevention program upon their request. The principal investigator held the prerogative to discontinue the entire trial, if new information that affected the benefit-to-risk ratio of the trial surfaced or if there was a recurrence of serious adverse events related to the trial. No such adverse events occurred during the trial and no participants needed to be excluded once they were randomized and active in the trial. Ethical approval was obtained by the Ethics Committee of the Medical School Berlin in March 2021 (MSB-2021/55).

### 2.2. Participants

#### 2.2.1. Inclusion Criteria

Participants were youth with care experience, that is, adolescents in foster or institutional care, adopted adolescents, or young care leavers aged between 14 and 21 years. This age range aligns with the definition for “adolescents” in the German Guidelines for Psychotherapy (article A, first paragraph, point 4). Furthermore, in Germany, adolescents up to the age of 21 years often remain dependent on foster or adoptive parents or reside in supervised institutionalized care. Therefore, the entire research consortium established a cut-off age of 21 years for inclusion. Additional inclusion criteria involve possessing adequate reading and writing skills in the German language, as determined by self-report, and having internet access via a desktop computer, laptop, tablet, or other mobile device.

#### 2.2.2. Exclusion Criteria

Participants were excluded from the RCT under certain circumstances, namely (1) suspected acute child endangerment, assessed via the Juvenile Victimization Questionnaire (JVQ) [18]; (2) receiving concurrent psychosocial interventions aimed at victimization or revictimization; (3) displaying severe depression symptoms, which was assessed using the Patient Health Questionnaire-9 for Adolescents (PHQ-9) [19] (specifically a score exceeding 15 at baseline); and (4) exhibiting suicidal ideation, denoted by a score exceeding 1 on the suicidal thought item of the PHQ-9 at baseline. In case of indications of acute child endangerment, severe depressive symptoms and instances where participants score above 1 on the suicide item, a thorough telephone screening was conducted in adherence to our safety protocol.

### 2.3. Internet-Based Prevention Program EMPOWER YOUTH

The EMPOWER YOUTH internet-based prevention program caters to the needs of youth in care, mitigating risk factors related to victimization [16]. Participants acquire theory and skills to prevent victimization in themselves and others. It aims to foster awareness of risk situations, their consequences, and strategies to protect youth from victimization (developed by B.W., B.M.v.N.). Upon program completion, participants earn an EMPOWER YOUTH coach certification, highlighting their role in helping others. This emphasis on coaching rather than victimization is essential to prevent potential stigma associated with living in care, which youth in care often experience in their everyday lives, as assessed in focus groups during program development. The intervention includes multimedia elements (videos, audio recordings, writing exercises) accessible through a personalized password-protected interface. It comprises six modules, each with around a 45-min workload. Psychologists in the role of mentors (B.M.v.N., O.L.M.E.) offered individual written feedback within two working days and guided participants to the next module, strengthening program compliance. Up to three reminders are sent to complete each module. Participants may choose to complete the modules at their own pace [16].

Derived from literature and insights from three focus groups with care-experienced adolescents and young adults, the six program modules address psychological risk factors: (1) emotion regulation, (2) self-appraisal, (3) risk recognition, (4) offline risk recognition and coping with victimization, and (5) online risk recognition and coping with victimization. Throughout these modules, interpersonal relatedness and prosocial behaviors are indirectly emphasized through case descriptions and exercises, some of which draw from real-life situations described by youth in care during the initial focus groups. The first module, “What’s up?”, focusses on information and exercises around emotion recognition and regulation. The second module, “I am okay, you are okay, we are okay”, deals with the topics of identity, personal rights, self-worth, and self-appraisal. In the third module, “Stop!”, participants are made familiar with and practice risk recognition. This is followed by learning and practicing how to set boundaries in offline risk situations in the fourth module, “Your limit”, and in online risk situations in the fifth module, “Like and share?”. The sixth module, “You’re the expert”, repeats the main lessons from the previous modules. More detailed module information can be found elsewhere [16,20].

### 2.4. Outcome Measures

#### 2.4.1. Primary Endpoint

The primary outcome consists of the change in frequency in victimization experiences, as assessed via the JVQ [18] and the Bullying Screener (BS) [21] for cyber-bullying as a victimization experience. The JVQ, a validated screening questionnaire, encompasses 34 offenses against youth across five areas: (1) conventional crime, (2) child maltreatment, (3) peer and sibling victimization, (4) sexual victimization, and (5) witnessing and indirect victimization. Two items (physical abuse by caregiver; witnessing a parent assault a sibling) were adapted to German law by not excluding spanking. Item 26 (statutory rape) inquires about consensual sexual activity with a person above the age of 18. Since consensual sex is legal from the age of 14 in Germany, it cannot be definitively classified as sexual victimization within this age group and was therefore excluded. Participants indicate whether they encountered each event in the past three months, with “yes” (1) or “no” (0) dichotomous responses. These scores are tallied to obtain a total score, with higher values indicating greater victimization exposure [18]. A score of ≥4 over a period of one year is indicative of poly-victimization, and ≥7 suggests high poly-victimization [22]. The items include an ordinal follow-up question to assess event frequency; for the current RCT, the time period is altered to an assessment of the past three months, ranging from “never” (0), “rarely (1–3 occurrences)” (1), “often (more than 4 occurrences)” (2), “very often (at least 1 occurrence per week)” (3). The frequency scores were totaled overall and per module [18]. The JVQ has been shown to have adequate test–retest reliability (κ = 0.63) and high internal consistency (Cronbach α = 0.80) [18]. Cronbach α in the current sample was excellent, at 0.90. The BS comprises six items that assess both victimization and perpetration of bullying behaviors. Respondents were queried about the frequency of these experiences over the past three months, with response options ranging from “never” to “a lot” (at least once a week) [21]. In this RCT, solely the items for cyber-bullying are of interest, from a victim perspective. Since this specific type of victimization is not included in the JVQ used for this study.

#### 2.4.2. Secondary Endpoint

Secondary outcomes encompassed risk perception, aggressive tendencies, empathy, prosocial behaviors, loneliness, depressiveness, and post-traumatic stress symptoms. The Adolescent Risk-taking Questionnaire (ARQ) [23] gauges risk-taking behaviors and perception. It consists of two sections. The first assesses adolescents’ riskiness judgments for 22 behaviors, rated on a 5-point Likert scale from “0” (not risky) to “4” (extremely risky). The second section evaluates the frequency of engaging in these behaviors, also on a 5-point Likert scale from “0” (never done) to “4” (done very often). In the present study, only the risk perception section was analyzed, as the risk behavior subscale contained items whose engagement by youth with care experience seemed rather infrequent (e.g., skydiving). Total risk perception as calculated, with higher scores indicating stronger judgments of riskiness. The items are categorized into four major factors: thrill-seeking behaviors (e.g., skip school, martial arts), rebellious behaviors (e.g., smoke cigarettes, take drugs), reckless behaviors (e.g., drink and drive, car racing), and antisocial behaviors (e.g., cheat, talk to strangers). The questionnaire demonstrates good reliability with Cronbach α coefficients ranging from 0.86 to 0.96 for the judgment scale. Test-retest reliability over one week is also strong, with coefficients of 0.79 for risk judgment [23].

Empathy, prosocial behavior, and aggression tendencies were assessed via three of the four subscales of the German Questionnaire for Determining Empathy, Prosocial Behavior, and Aggression (FEPAA) [24]. Two different versions (A and B) of the questionnaire were employed at the two assessment points. Cronbach α ranged from 0.61 (prosocial behavior scale) to 0.79 (aggressive tendencies scale) for version A and from 0.57 (prosocial behavior scale) to 0.77 (aggressive tendencies scale) for version B. Reliability is 0.75 for version A and 0.66 for version B in the original validation study [24]. In the current study, a total of 40 items out of the original 55 were assessed. Cronbach α reached acceptable levels of 0.65 for the prosocial behavior scale and 0.66 for the aggressive tendencies scale. The names in the FEPAA items were changed to more modern und more diverse names, with official approval of the publisher. Standard scores were derived separately for each gender, with scores between 90 and 110 being average.

Loneliness was measured using the Loneliness Scale-SOEP [25], consisting of three items with a 5-point rating scale. The reliability coefficient (α) obtained was 0.80.

Depressiveness was evaluated using the PHQ-9 [19], which screens for the presence and severity of depression. This assessment takes less than 3 min to complete. Item 9 specifically assesses the presence of suicidal and/or self-harm thoughts. The PHQ-9 demonstrated a Cronbach α of 0.89 among 3000 primary care patients and a test–retest reliability of 0.84 for in-person and phone interviews with the same patients [19]. In the current study, a Cronbach α of 0.93 was reached, indicating an excellent internal consistency. The sum score reflects different levels of depression severity: 0–4 none, 5–9 mild, 10–14 moderate, 15–19 moderately severe, 20–27 severe depressiveness [19].

The presence of post-traumatic stress symptoms was assessed using the 8-item version of the Child Revised Impact of Events Scale (CRIES-8) [26]. This scale utilizes a 4-point rating scale and takes between 5 and 10 min to complete. A total score, as well as intrusive thoughts and avoidance behavior, is assessed. A clinical cut-off sum score of 17 or greater correctly identifies children with a diagnosis of post-traumatic stress disorder with a sensitivity of 0.94 and specificity of 0.59 [26]. Internal consistency was good, with a Cronbach α of 0.83 for the intrusion subscale, 0.79 for the avoidance subscale, and 0.88 for the sum score.

### 2.5. Statistical Analyses

The primary outcome is change in occurrence of victimization experiences in the previous three months (JVQ sum score and BS subscale cyber-bullying) from baseline to the 18-week follow-up. Secondary outcomes encompass risk perception on four subscales (ARQ), aggressive tendencies (FEPAA), empathy (FEPAA), prosocial behavior (FEPAA), loneliness (LS-S), depressiveness (PHQ-9 sum score), and post-traumatic stress symptoms (CRIES-8 subscales and sum score). Statistical analyses were performed in IBM SPSS 27 [27]. Demographic variables were analyzed descriptively. No extreme outliers (>3 standard deviations from the mean) were identified upon boxplot inspection in the outcome variables. The continuous variables demonstrated a normal distribution based on Q–Q plot inspection, except for the following variable: ‘recklessness perception’ (ARQ subscale) both at baseline and follow-up. One-way ANOVAs examined the differences between the two groups at baseline. A completer intention-to-treat design was employed for efficacy analyses. Repeated measures ANOVAs examined the primary and secondary endpoints, with ‘intervention group’ and ‘assessment time point’ as between- and within-factors. The categorical secondary endpoints were assessed using chi-square tests or Fisher exact tests. To predict intervention non-starters and dropouts, a multinomial logistic regression analysis was run. The following baseline characteristics were considered as prediction factors in the model: age (in years), time spent in the care system (in years), the sum of different lifetime victimization experiences (lifetime JVQ sum score), the level of depressiveness at baseline (PHQ-9 sum score), and the level of post-traumatic stress symptoms at baseline (CRIES-8 sum score). For all tests, a significance level of 5% was used.

## 3. Results

### 3.1. Sample Description

**Figure 1:**
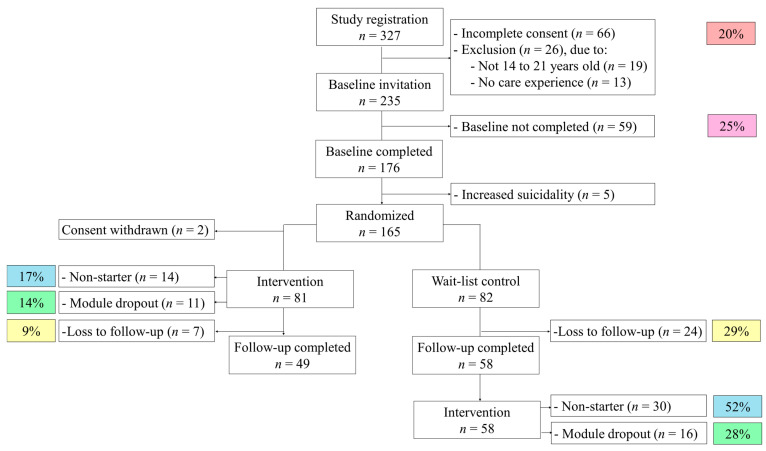
Study flowchart including dropout rates.

A total of 327 participants registered for the study, of which 176 participated in the baseline assessment and 165 participants could be randomized and included in the trial. Exclusion reasons can be found in the study flowchart (see Figure 1). As two participants retracted their study consent right after randomization, 163 participants started the trial. The average age at baseline of these 163 participants was 17.68 years (*SD* = 2.11, age range = 14 to 21 years). A total of 68 percent of participants identified as female (*n* = 110), 4 percent identified as divers (neither male nor female; *n* = 7). Most participants (88 percent, *n* = 143) were born in Germany. The other participants were born in Austria (*n* = 9), Ukraine (*n* = 2), England (*n* = 1), Italy (*n* = 1), Poland (*n* = 1), Vietnam (*n* = 1), Iran (*n* = 1), Eritrea (*n* = 1), Paraguay (*n* = 1), Uzbekistan (*n* = 1), and unknown to the participant (*n* = 1). The majority of participants, 65% (*n* = 106), were living in an institutionalized care setting at the time of assessment, 23 participants (14%) were living in a foster family, 10 in an adoptive family, (6%) 4 in kinship care (3%), 7 participants (4%) were currently living with their biological families again, after care experience in the past, and 13 participants (8%) were living on their own, so-called care leavers. Participants reported to have care experience for an average of 6.95 years (*SD* = 5.49, range = 2 months to 20 years). A total of 42 percent of participants (*n* = 69) received psychotherapeutic treatment at the time of the baseline assessment. No serious adverse events or adverse events in general arose during the trial.

To achieve 80% statistical power, a sample size of 156 subjects at follow-up was deemed necessary [16]. With a total of *n* = 107 participants at follow-up, 69% of this sample size was achieved. The intervention efficacy is calculated with participants who completed the baseline and the follow-up assessment, see Figure 1. The baseline characteristics of this completer intention-to-treat sample can be found in Table 1.

### 3.2. Main Results

The victimization frequency measured with the JVQ and BS has not significantly changed in the intervention arm compared to the wait-list control group from baseline to follow-up (*p* > 0.050; see Table 2). However, over time, the frequency of victimization experiences is significantly reduced in both study groups (*p* = 0.012, η_p_^2^ = 0.06; *p* = 0.017, η_p_^2^ = 0.06, respectively).

The intervention group significantly increased their perception of thrill-seeking risks and rebellious risks, as shown by the significant interaction of group and time-point (*p* = 0.036, η_p_^2^ = 0.04; *p* = 0.026, η_p_^2^ = 0.05 respectively). The risk perception of reckless risks also increased in the intervention group; however, it did not reach significance (*p* = 0.061, η_p_^2^ = 0.03). Regarding perception of social risks, no significant interaction could be found (*p* = 0.990, η_p_^2^ = 0.00).

Over time, aggressive tendencies increased in both the intervention and wait-list groups (*p* < 0.001, η_p_^2^ = 0.67). However, for aggressive tendencies, no main effect for group (*p* = 0.225, η_p_^2^ = 0.01) and no interaction between group and time was found (*p* = 0.089, η_p_^2^ = 0.03). On average, the aggressive tendencies were in the average range and not clinically heightened or decreased.

Similarly, empathy and prosocial behavior both increased over time in both groups (*p* < 0.001, η_p_^2^ = 0.19; *p* < 0.001, η_p_^2^ = 0.74, respectively). For empathy, neither a main effect for group (*p* = 0.564, ηp2 = 0.00) nor a significant interaction effect was observed (*p* = 0.474, η_p_^2^ = 0.01). Similar for prosocial behavior, there was no main effect for group (*p* = 0.736, η_p_^2^ = 0.00) and no interaction effect (*p* = 0.146, η_p_^2^ = 0.02) found. Empathy ability and prosocial behaviors were on a group average level both in the average range at baseline and follow-up and were neither heightened nor decreased.

For loneliness, no statistically significant interaction between group and time was found (*p* = 0.492, η_p_^2^ = 0.01), and no main effects for group (*p* = 0.885, η_p_^2^ = 0.00) or time (*p* = 0.267, η_p_^2^ = 0.01) were found either. Similarly, for depressive symptoms, no significant interaction was observed (*p* = 0.756, η_p_^2^ = 0.00), nor effects of group (*p* = 0.826, η_p_^2^ = 0.00) or time (*p* = 0.050, η_p_^2^ = 0.04). At baseline, the clinical cut-off for moderate depressiveness was reached by 43% of the intervention group and 47% of the wait-list control group. More specifically, 14% of the intervention arm and 16% of the control group reached self-report levels of severe depressiveness at baseline.

For post-traumatic stress symptoms, i.e., intrusions, avoidance, and a sum score, neither a main effect for group (*p* = 0.791, η_p_^2^ = 0.01; *p* = 0.685, η_p_^2^ = 0.00; *p* = 0.359, η_p_^2^ = 0.01, respectively) nor a main effect for time (*p* = 0.571, η_p_^2^ = 0.01; *p* = 0.789, η_p_^2^ = 0.00; *p* = 0.760, η_p_^2^ = 0.00, respectively) nor an interaction effect between group and time could be found from baseline to follow-up (*p* = 0.376, η_p_^2^ = 0.01; *p* = 0.944, η_p_^2^ = 0.00; *p* = 0.729, η_p_^2^ = 0.00, respectively). Clinical levels of post-traumatic stress symptoms are reported by 59% of participants of the intervention group and 64% of the wait-list group. This coincides with reports of receiving psychotherapeutic treatment by 42% and 43%, respectively.

### 3.3. Attrittion and Dropout Analyses

Of the 81 participants who were invited to the internet-based prevention program directly after baseline, 14 (17%) did not start or complete the first module, so-called non-starters. During the intervention, another 11 (14%) did not finish all the modules and dropped out during the intervention (see Figure 1). A total of 56 participants completed the intervention in the intervention group (69%). A total of 7 participants did not participate in the follow-up assessment (7%). In the wait-list control group, there is a loss to follow-up of 24 participants from the original 82 participants (29%). The remaining participants in the control group (*n* = 58) were invited to partake in the intervention, after study completion and after they received study compensation. A total of 30 participants did not start the intervention (52%) and 16 participants dropped out during the intervention (28%). A total of 12 participants of the wait-list control group completed the intervention (21%). 

The attrition rate for both study arms was significantly different (*χ*^2^ = 32.293, *p* =< 0.001), with significantly less intervention completers in the wait-list control group compared to the intervention group. Descriptive baseline variables were compared between intervention completers (*n* = 68), non-starters (*n* = 44), and module dropouts (*n* = 27), regardless of study arm. The following variables were not normally distributed and non-parametric Kruskal–Wallis H tests were conducted instead of one-way ANOVAs due to the smaller sample size: ‘duration of care experience’, ‘depressiveness’ (PHQ-9 sum score), and ‘post-traumatic stress symptoms’ (CRIES-8 sum score). No significant differences between intervention completers, non-starters, and dropouts during the intervention were found in the descriptive variables listed in Table 3. The multinomial logistic regression model was not statistically significant either (*χ*^2^(10) = 4.837, Nagelkerke *R*^2^ = 0.040, *p* = 0.902). None of the selected baseline variables reliably predicted attrition type.

## 4. Discussion

The current study aimed to evaluate the efficacy of the newly developed internet-based prevention program EMPOWER YOUTH. The program did not reduce the occurrence of victimization experiences in those who received the intervention compared to the wait-list control group (primary objective). However, victimization experiences decreased over time in both groups. Similarly, the program did not affect aggressive tendencies, empathy, prosocial behavior, loneliness, depression, or post-traumatic stress symptoms (secondary objectives). The intervention showed a significant increase in risk perception of thrill-seeking and rebellious risk behaviors. The perception and recognition of reckless and antisocial risk behaviors did not change.

The current sample of youth with care experience reports high levels of poly-victimization [22], especially for lifetime victimization. At the same time, no cut-offs were defined for shorter periods, such as three months, making a definite interpretation of the number of victimization experiences limited. Despite the clear presence of victimization experiences in care experiences in youth, the EMPOWER YOUTH program appears to have no effect on their occurrence. One possible explanation is the relatively low variance in the number of victimization experiences at baseline and follow-up, which was perpetuated by the adapted and shortened assessment period of three months versus six months [18]. 

The EMPOWER YOUTH program significantly affected aspects of risk perception, aligning with the intervention modules’ content and their clear focus on risk perception [16]. However, despite the fact that impaired risk recognition increases the likelihood of revictimization [8,11], the program might not have sufficiently addressed other mechanisms that increase victimization risk, such as interpersonal difficulties [9,10], low self-esteem [10], impaired emotion regulation [10], and increased threat of harm perception [9]. Therefore, focusing primarily on risk perception is not sufficient to change the number of victimization experiences. In addition to changes in risk recognition and heightened awareness of the potential risks associated with thrill-seeking and rebellious behaviors, other factors in the everyday environment of youth with care experience play a decisive role.

Some categories of risk behavior were perceived as more risky after completing the program. However, the risks associated with reckless and antisocial behaviors were not viewed differently after the EMPOWER YOUTH program. The antisocial behaviors listed are a mix of several different actions, such as cheating, talking to strangers, and overeating, which makes it less likely that the intervention can have a targeted influence on this specific category. Most of the listed reckless behaviors were associated with cars and driving, a topic more familiar to youth in the United States, where the questionnaire was developed, than to youth in Germany. Despite this, reckless behaviors such as drinking and driving or car racing were clearly perceived as having the highest potential risk compared to the other three categories of risk behaviors. Therefore, an increased perception of their risk potential by the program may not be necessary in the first place.

In addition to the high levels of poly-victimization observed in the current sample of care-experienced youth, almost half of the sample report high levels of depressive symptoms, and more than half report clinically relevant post-traumatic stress symptoms, which is in line with previous literature [3,4,5,6]. No reduction in these symptoms was found, which can be explained by the fact that the intervention was neither trauma-specific nor aimed at treating depressive symptoms specifically. This large subgroup of participants suffering from mental disorders might not benefit from a prevention program that solely targets victimization experiences without simultaneously addressing their psychopathology. For these particular care-experienced youth, a prevention program alone seems insufficient to decrease PTSD and depressive symptoms significantly. Instead, this internet-based prevention program should be regarded as an add-on method, offered alongside other interventions that target existing psychopathology rather than focusing solely on prevention and/or victimization.

Moreover, the internet-based setting might not be beneficial for all participants, despite the low-threshold accessibility of an online program. Specifically for youth with care experience, a new face-to-face intervention in a group setting in institutionalized care has been developed, which has shown promising initial results in a pre-post-treatment design [28]. This group intervention aims to address critical life events and adverse childhood experiences through life story work (biographical work) [28]. However, a randomized controlled trial of this intervention should be still conducted.

The dropout rate of 14% after actively starting the intervention is lower than the expected 30%, which was based in part on the pilot feasibility and usability study [29]. The low dropout rate during active participation in the intervention aligns with previously reported positive usability ratings of the program [29]. Another explanation for the lower dropout rate is the individualized written feedback by psychologists, which was implemented to increase compliance. The highest completion rates for e-mental health programs for children, adolescents, and young adults are indeed found in programs incorporating therapist support, with dropout rates ranging from 13% to 17% [30].

While the dropout rate during the intervention was notably low, the overall RCT still suffered from several dropouts at various points during the trial. Participants’ study registration and successful completion of the baseline assessment do not guarantee actual participation in the study trial. Many of these dropouts are explained by challenges in the study design; for instance, youth younger than 16 years needed additional consent from a legal guardian. In 20% of cases, the consent forms were incomplete, preventing the youth from participating in the study.

## 5. Conclusions

Overall, the internet-based prevention program EMPOWER YOUTH effectively increased the risk perception of thrill-seeking and rebellious behaviors. This altered risk perception can potentially have a positive impact on the everyday lives of youth with care experience. However, the number of victimization experiences in the intervention group could not be significantly reduced compared to the waiting-list control group, which was the primary objective. About half of the youth with care experience participating in this trial exhibited increased levels of psychopathological symptoms. Therefore, prevention programs aimed at this highly vulnerable group of youth in care should be considered an add-on to more intensive interventions focused on common mental health disorders in this group (e.g., PTSD, depression), rather than as a stand-alone online program. In line with this, approximately 42% of the participants were receiving face-to-face psychotherapeutic treatment at the time of the trial. Rather than solely focusing on revictimization, there is a strong need for low-threshold access to effective interventions for psychopathology, e.g., in the form of specialized treatment centers.

## Figures and Tables

**Table 1:** Sample description of the completer sample at baseline assessment.

	Intervention (*n* = 81)	Wait-List (*n* = 82)	*t*-Test/ Chi-Square Test		
	*M ± SD/n* (%)	*M ± SD/n* (%)	*p*	*d*	*φ*
Age (in years)	17.47 ± 2.06	17.90 ± 2.16	0.195	0.20	-
Gender (female)	50 (62%)	60 (73%)	0.226	-	0.14
Current living situation			0.519	-	0.18
Institutionalized care	52 (64%)	54 (66%)			
Foster care	10 (12%)	13 (16%)			
Adoption	8 (10%)	2 (3%)			
Kinship care	2 (3%)	2 (2%)			
Biological parent	3 (4%)	4 (5%)			
Independent (care leaver)	6 (7%)	7 (9%)			
Duration of care experience (in years)	7.21 ± 5.18	6.79 ± 5.84	0.627	0.08	-
Receiving psychotherapeutic treatment	34 (42%)	35 (43%)	0.927	-	0.01
Lifetime victimization (JVQ sum score)	12.33 ± 6.20	12.44 ± 6.84	0.914	0.02	-
Lifetime victimization type (≥1 item)					
Conventional crime	79 (98%)	78 (96%)	0.650	-	0.04
Child maltreatment	73 (90%)	71 (88%)	0.617	-	0.04
Peer victimization	67 (83%)	63 (78%)	0.430	-	0.06
Sexual victimization	47 (58%)	57 (70%)	0.101	-	0.13
Witnessing violence	66 (82%)	64 (79%)	0.693	-	0.03

Note. JVQ = Juvenile Victimization Questionnaire, cut-off high poly-victimization ≥ 7 [18]; *d* = Cohen’s *d* effect size, small = 0.20, medium = 0.50, and large = 0.80; *φ* = Phi’s coefficient effect size, small = below 0.29, medium = 0.30 to 0.49, and large = 0.50 or greater.

**Table 2:** Efficacy results (ANOVA) with primary and secondary outcomes for the intervention (*n* = 49) and wait-list control group (*n* = 58).

	Baseline		Follow-Up		Group		Time-Point		Group × Time	
	Intervention *M* ± *SD*	Wait-List *M* ± *SD*	Intervention *M* ± *SD*	Wait-List *M* ± *SD*	*p*	η_p_^2^	*p*	η_p_^2^	*p*	η_p_^2^
Primary outcome (previous 3 months)										
Victimization frequency (JVQ)	5.95 ± 7.68	3.91 ± 5.26	4.45 ± 6.83	2.98 ± 4.90	0.114	0.37	**0.012**	0.06	0.546	0.00
Cyber-bullying victim frequency (BS)	0.34 ± 0.68	0.20 ± 0.41	0.11 ± 0.54	0.04 ± 0.30	0.114	0.02	**0.017**	0.06	0.649	0.00
Secondary outcome										
Risk perception (ARQ)										
Thrill-seeking risks	1.28 ± 0.70	1.38 ± 0.63	1.45 ± 0.62	1.30 ± 0.60	0.796	0.00	0.478	0.01	**0.036**	0.04
Rebellious risks	2.58 ± 0.79	2.76 ± 0.77	2.84 ± 0.64	2.72 ± 0.71	0.752	0.00	0.091	0.03	**0.026**	0.05
Reckless risks	3.18 ± 0.86	3.38 ± 0.66	3.37 ± 0.76	3.34 ± 0.53	0.485	0.01	0.198	0.02	0.061	0.03
Antisocial risks	2.18 ± 0.92	2.30 ± 0.75	2.19 ± 0.78	2.31 ± 0.72	0.371	0.01	0.839	0.00	0.990	0.00
Aggressive tendency (FEPAA)	104.45 ± 7.60	104.72 ± 6.51	90.67 ± 10.67	93.88 ± 9.96	0.225	0.01	**<0.001**	0.67	0.089	0.03
Empathy (FEPAA)	101.55 ± 9.22	101.56 ± 10.21	96.71 ± 8.19	95.07 ± 9.51	0.564	0.00	**<0.001**	0.19	0.474	0.01
Prosocial behavior (FEPAA)	107.88 ± 12.21	110.09 ± 11.80	89.04 ± 5.72	87.77 ± 5.75	0.736	0.00	**<0.001**	0.74	0.146	0.02
Loneliness (LS-S)	5.85 ± 3.67	5.59 ± 3.28	4.98 ± 3.37	5.38 ± 3.36	0.885	0.00	0.267	0.01	0.492	0.01
Depressiveness (PHQ-9)	10.71 ± 7.78	11.17 ± 7.77	9.85 ± 7.41	10.00 ± 7.27	0.826	0.00	0.050	0.04	0.756	0.00
Post-traumatic stress symptoms (CRIES-8)										
Intrusions	7.82 ± 5.66	9.32 ± 6.36	8.08 ± 6.43	8.63 ± 6.15	0.791	0.01	0.685	0.00	0.359	0.01
Avoidance	9.45 ± 5.85	10.11 ± 6.12	9.43 ± 6.64	10.42 ± 6.45	0.571	0.01	0.789	0.00	0.760	0.00
Sum score	17.27 ± 10.80	19.42 ± 11.42	17.51 ± 12.19	19.05 ± 11.90	0.376	0.01	0.944	0.00	0.729	0.00

Note. Bold indicates significant results; JVQ = Juvenile Victimization Questionnaire [18]; BS = Bullying Screener [21]; ARQ = Adolescent Risk Questionnaire [23]; FEPAA = Questionnaire for Empathy, Prosocial Behavior, and Aggression, average scores >90 <110 [24]; LS-S = Loneliness Scale-SOEP [25]; PHQ-9 = Patient Health Questionnaire 9-item, moderate depressiveness >10, moderately severe >15, severe depressiveness >20 [19]; CRIES-8 = Children’s Revised Impact of Event Scale 8 item, sum score cut-off ≥ 17 [26]; η_p_^2^ = eta-squared; effect size small = 0.01, medium = 0.06, large = 0.14.

**Table 3:** Comparison between intervention completers (*n* = 68), non-starters (*n* = 44), and module dropouts (*n* = 27).

	Completers	Intervention Non-Starters	Module Dropout	ANOVA/Kruskal-Wallis H-Test/ Chi-Square Test		
	*M* ± *SD*/*n* (%)	*M* ± *SD*/*n* (%)	*M* ± *SD*/*n* (%)	*p*	η^2^	*φ*
Age (in years)	17.62 ± 2.06	17.77 ± 2.15	17.84 ± 2.20	0.859	0.01	-
Gender (female)	45 (66%)	35 (80%)	17 (63%)	0.491	-	0.16
Current living situation				0.303		0.32
Institutionalized care	42 (62%)	28 (64%)	18 (67%)			
Foster care	8 (12%)	6 (14%)	4 (15%)			
Adoption	8 (12%)	1 (2%)	1 (4%)			
Kinship care	1 (2%)	1 (2%)	2 (7%)			
Biological parent	2 (3%)	5 (11%)	0 (0%)			
Independent (care leaver)	7 (10%)	3 (7%)	2 (7%)			
Duration of foster care	7.20 ± 5.40	6.20 ± 5.07	7.42 ± 5.94	0.602	0.01	-
Receiving psychotherapy	34 (50%)	14 (32%)	11 (41%)	0.161	-	0.16
Lifetime victimization (JVQ)	11.89 ± 6.19	13.07 ± 6.50	14.15 ± 6.29	0.215	0.02	-
Depressiveness (PHQ-9)	10.45 ± 7.68	11.43 ± 7.80	10.93 ± 7.49	0.748	0.01	-
Post-traumatic stress (CRIES)	17.84 ± 11.27	19.95 ± 11.24	20.19 ± 10.86	0.511	0.01	-

Note. JVQ = Juvenile Victimization Questionnaire, cut-off high poly-victimization ≥ 7 [18]; PHQ-9 = Patient Health Questionnaire 9-item, cut-off moderate depressiveness >10, moderately severe >15, severe depressiveness >20 [19]; CRIES-8 = Children’s Revised Impact of Event Scale 8-item, cut-off ≥ 17 [26]; η^2^ = eta-squared; effect size small = 0.01, medium = 0.06, large = 0.14; *φ* = Phi’s coefficient effect size, small = below 0.29, medium = 0.30 to 0.49, and large = 0.50 or greater.

## Data Availability

The data set is not available anymore due to the data regulation limitations of the data regulation board and the ethics committee in accordance with the General Data Protection Regulation (GDPR) in Europe and Germany. The corresponding author can be contacted for further information on the data set.
